# Identification and evaluation of risk of generalizability biases in pilot versus efficacy/effectiveness trials: a systematic review and meta-analysis

**DOI:** 10.1186/s12966-020-0918-y

**Published:** 2020-02-11

**Authors:** Michael W. Beets, R. Glenn Weaver, John P. A. Ioannidis, Marco Geraci, Keith Brazendale, Lindsay Decker, Anthony D. Okely, David Lubans, Esther van Sluijs, Russell Jago, Gabrielle Turner-McGrievy, James Thrasher, Xiaming Li, Andrew J. Milat

**Affiliations:** 1 Arnold School of Public Health, University of South Carolina, Columbia, SC USA; 2 Departments of Medicine, of Health Research and Policy, of Biomedical Data Science, and of Statistics, and Meta-Research Innovation Center at Stanford (METRICS), Stanford University, Stanford, CA USA; 3 Early Start, Faculty of Social Sciences, University of Wollongong, Wollongong, NSW Australia; 4 Priority Research Centre in Physical Activity and Nutrition, School of Education, University of Newcastle, Callaghan, New South Wales Australia; 5 Centre for Diet and Activity Research & MRC Epidemiology Unit, School of Clinical Medicine, University of Cambridge, Cambridge, UK; 6 Centre for Exercise Nutrition & Health Sciences, School for Policy Studies, University of Bristol, Bristol, UK; 7 New South Wales (NSW) Ministry of Health, St Leonards, NSW Australia; 8 Sydney Medical School, The University of Sydney, Sydney, Australia

**Keywords:** Intervention, Childhood obesity, Youth, Physical activity, Sleep, Diet, Screen time, Scalability, Framework

## Abstract

**Background:**

Preliminary evaluations of behavioral interventions, referred to as pilot studies, predate the conduct of many large-scale efficacy/effectiveness trial. The ability of a pilot study to inform an efficacy/effectiveness trial relies on careful considerations in the design, delivery, and interpretation of the pilot results to avoid exaggerated early discoveries that may lead to subsequent failed efficacy/effectiveness trials. “Risk of generalizability biases (RGB)” in pilot studies may reduce the probability of replicating results in a larger efficacy/effectiveness trial. We aimed to generate an operational list of potential RGBs and to evaluate their impact in pairs of published pilot studies and larger, more well-powered trial on the topic of childhood obesity.

**Methods:**

We conducted a systematic literature review to identify published pilot studies that had a published larger-scale trial of the same or similar intervention. Searches were updated and completed through December 31st, 2018. Eligible studies were behavioral interventions involving youth (≤18 yrs) on a topic related to childhood obesity (e.g., prevention/treatment, weight reduction, physical activity, diet, sleep, screen time/sedentary behavior). Extracted information included study characteristics and all outcomes. A list of 9 RGBs were defined and coded: intervention intensity bias, implementation support bias, delivery agent bias, target audience bias, duration bias, setting bias, measurement bias, directional conclusion bias, and outcome bias. Three reviewers independently coded for the presence of RGBs. Multi-level random effects meta-analyses were performed to investigate the association of the biases to study outcomes.

**Results:**

A total of 39 pilot and larger trial pairs were identified. The frequency of the biases varied: delivery agent bias (19/39 pairs), duration bias (15/39), implementation support bias (13/39), outcome bias (6/39), measurement bias (4/39), directional conclusion bias (3/39), target audience bias (3/39), intervention intensity bias (1/39), and setting bias (0/39). In meta-analyses, delivery agent, implementation support, duration, and measurement bias were associated with an attenuation of the effect size of − 0.325 (95CI − 0.556 to − 0.094), − 0.346 (− 0.640 to − 0.052), − 0.342 (− 0.498 to − 0.187), and − 0.360 (− 0.631 to − 0.089), respectively.

**Conclusions:**

Pre-emptive avoidance of RGBs during the initial testing of an intervention may diminish the voltage drop between pilot and larger efficacy/effectiveness trials and enhance the odds of successful translation.

## Background

Pilot testing of behavioral interventions (aka feasibility or preliminary studies) is a common part of the process of the development and translation of social science/public health interventions [1–6]. Pilot studies, within the translational pipeline from initial concept to large-scale testing of an intervention, are conducted to *“provide information of high utility to inform decisions about whether further testing [of an intervention] is warranted* [7].*”* In pilot studies, preliminary evidence on feasibility, acceptability, and potential efficacy of an intervention are collected [1–5]. Across major government funders, such as the National Institutes of Health (NIH), the Medical Research Council and National Institute of Health Research in the United Kingdom, the National Health and Medical Research Council of Australia, and the Canadian Institutes of Health Research, pilot studies play a prominent role in the development and funding of almost all large-scale, efficacy/effectiveness intervention trials. This is evidenced by funding mechanisms specifically for pilot studies (e.g., NIH R34) [7], the requirement of preliminary data presented in grant applications, and the inclusion of pilot studies as a key stage in the development and evaluation of complex interventions [8].

Pilot studies have received heightened attention over the past two decades. This attention has focused on what constitutes a pilot study, the type of information a pilot study can and cannot provide, whether hypothesis testing is or is not appropriate within a pilot study, the various research designs one could employ, and debates about their proper nomenclature [1–6, 9–13]. More recently, peer-reviewed scientific journals have been created with a specific focus on pilot studies, as well as an extension to the CONSORT Statement focusing on various aspects of reporting pilot/feasibility studies [9]. These articles raise important considerations in the conduct and reporting of pilot studies, and decision processes regarding whether or not to proceed with a large-scale, efficacy/effectiveness trial, yet they focus largely on topics related to threats to internal validity that may ensue.

Biases can lead to incorrect conclusions regarding the true effect of an intervention, and can be introduced anywhere along the translational pipeline of behavioral interventions – from the initial development and evaluation during a pilot study, in the large-scale randomized efficacy or effectiveness trial, to the evaluation of an intervention in a dissemination and implementation study [14, 15]. Biases relevant to internal validity, such as whether blinding or randomization were used, rates of attrition, and the selective reporting of outcomes [16] are important considerations when designing an intervention trial or evaluating published studies. However, intervention researchers need to also consider external validity in the design, conduct, and interpretation of pilot studies. The introduction of biases related to external validity can lead to prematurely scaling-up an intervention for evaluation in a larger, efficacy/effectiveness trial.

Internal validity deals with issues related to whether the receipt of the intervention was the cause for change in the outcome(s) of interest in the specific experimental context under which an intervention was tested [17]. In contrast, external validity refers to the variations in the conditions (e.g., target audience, setting) under which the intervention would exhibit the same or similar impact on the outcome(s) of interest [17]. These are important distinctions, as the vast majority of checklists for the design and conduct of a study focus on topics related to internal validity, as noted by the widely endorsed risk of bias checklists [16] and trial reporting statements [18, 19], while largely ignoring whether the casual inference, in this case the inference drawn from a pilot study, are likely to generalize to variations in study conditions that could occur in a larger-scale, more well-powered trial. Thus, if the purpose of conducting pilot studies is to “*inform decisions about whether further testing [of an intervention] is warranted* [7]”, it is then reasonable to expect a great deal of emphasis would be placed on aspects of external validity, particularly when determining if a larger-scale trial is necessary.

### Rationale of the proposed “risk of generalizability biases”

Biases related to external validity present in a pilot study can result in misleading information about whether further testing of the intervention, in a larger, efficacy/effectiveness trial, is warranted. We define **“risk of generalizability biases”** as the degree to which features of the intervention and sample in the pilot study are NOT scalable or generalizable to the next stage of testing in a larger, efficacy/effectiveness trial. We focus on whether aspects like who delivers an intervention, to whom it is delivered, or the intensity and duration of the intervention during the pilot study are sustained in the larger, efficacy/effectiveness trial. The use of the term “bias” in this study therefore refers to ways in which features of the pilot study lead to systematic underestimation or overestimation of the assessment regarding the viability of the tested intervention and, subsequently, influence the decision whether to progress to the next stage of evaluating the intervention in a larger, more well-powered trial is necessary.

There is a history of studies that have evaluated the same (or very similar) interventions yet produce different outcomes when conducted under efficacy or effectiveness conditions, a phenomenon referred to as “voltage drop” [20–23]. Conducting a study from an efficacy perspective may ignore important aspects of generalizability that are associated with the design and conduct of an effectiveness study [24]. Doing so can introduce external validity biases (either knowingly or unknowingly) that may change the effect the intervention has on outcomes. In Table 1, we present examples from a sample of six interventions [25–30, 32–37] related to childhood obesity that have a published efficacy and a subsequent effectiveness trial and one intervention [31] with only an efficacy evaluation published. In these studies [25–37], the authors indicate the substantially reduced or null effects observed in the effectiveness trial may be due to a feature of the efficacy study, such as delivery of the intervention by study personnel, being removed in the effectiveness trial [38]. These are but a few of the adaptations interventionists could make [39] that may lead to possible biases that distort the estimated impact of an intervention, especially during pilot testing.

**Table 1:** Examples of Generalizability Biases in the Childhood Obesity Literature

Bias	Likely Larger Effect	Likely Smaller/No Effect
Study	Fitzgibbon 2005 [25]	Kong 2016 [26]
Who delivered the intervention?	“…the use of specially trained early childhood educators rather than classroom teachers to deliver the intervention, thereby raising questions of generalizability.”	“…using teachers in existing Head Start classrooms to deliver the intervention.”
Study	Cohen 2015 [27]	Sutherland 2017 [28]
How much of the intervention was provided?	1 full day training and 1 half day training	1 90-min training
Study	Beets 2016 [29]	Beets 2018 [30]
How much support to implement the intervention was provided?	“During the first year of receiving the intervention for both the immediate and delayed program, each program received four booster sessions. During the second year of receiving the intervention (for the immediate condition only) 2 booster sessions/program were provided.”	No additional onsite booster sessions or follow-up
Study	Sutherland 2016 [31]	
Who delivered the intervention?	“The provision of an in-school physical activity consultant for 1 day per week was the largest cost relating to the efficacy trial (66% of the total intervention cost). Whilst the provision of an in-school physical activity consultant was necessary under efficacy trial conditions in order to evaluate the effect of the combination of intervention strategies, the feasibility of providing a part-time consultant within schools across large geographic regions and the cost of such a model of support presents challenges in upscaling the intervention. The dissemination of an effective intervention across the community requires the use of implementation strategies which better mirror real world practice.”	
Study	McKenzie 1996 [32]	Hoelscher 2004 [33] (PE outcomes)
How much support to implement the intervention was provided?	“Following initial training, CATCH PE consultants provided on-site follow-up approximately every 2 weeks. During the 2.5 years, consultants made 3089 documented school visits, averaging 55.3 per school and 51.7 min in length. Consultants performed various roles during visits, including giving feedback to teachers, modeling new lesson segments, team teaching, and providing motivation and technical support.”	No onsite, on-going support provided
Study	Salmon 2008 [34]	Salmon 2011 [37]
How much of the intervention was provided?	19 lessons delivered	6 lessons delivered “…Switch-2-Activity involved an abbreviated programme; therefore, the intervention ‘dose’ was lower…”
How long was the intervention delivered?	10 months	7 weeks
Who delivered the intervention?	“All intervention components were delivered by one intervention specialist (a qualified Physical Education teacher) across all three schools.”	“the programme was delivered by regular class teachers rather than by a specialist university research team…”
What measures were used to collect information on outcomes?	Objective measures	Self-report
Study	West 2010 [35]	Gerards 2015 [36]
Who delivered the intervention?	“All sessions were facilitated by a clinical psychologist and accredited provider of the intervention (who co-authored the intervention materials), with assistance from graduate students in nutrition and dietetics, physical education, and psychology.”	“The intervention was led by three different facilitators. These health professionals have been accredited after attending an official 3-day training course and an additional intervention day.” “Finally, the West 2010 [35] study was implemented as an efficacy study, while in the current trial we tried to implement in the real life situation, which may have led to less significant study results.”
Who received the intervention?	“participants were mainly white, well-educated parents with moderate levels of employment and income.”	

Interventions that are pilot tested using highly skilled individuals, or extensive support for implementation, and/or short evaluations of the intervention may fail eventually if these features are not retained in the next phase of evaluation. Given pilot studies are often conducted with smaller sample sizes [40], it may be easier to introduce certain features, such as delivering the intervention by the researchers or providing extensive support for implementation, on a smaller scale than when testing an intervention in a larger trial that includes a larger sample size and more settings within which to provide the intervention. Pilot studies, therefore, may be more susceptible to introducing features that lead to underestimation or overestimation of an intervention’s viability for testing in a larger, more well-powered trial.

The definition of risk of generalizability biases, as applied to pilot intervention studies, is grounded in concepts within the scalability, scaling-up, and dissemination/implementation of interventions for widespread uptake and population health impact [39, 41–50] and pragmatic trial design [51–53]. The scalability literature describes key considerations interventionists must consider when taking an intervention that is efficacious “to scale” for population health impact. These include the human, technical and organizational resources, costs, intervention delivery and other contextual factors required to deliver the intervention and how the intervention interacts within the setting in which it is evaluated, such as schools that have close relationships with the research team, that may not be replicable in a larger study. These elements are consistent within implementation frameworks [20–22, 54–58], which describe the need to consider the authenticity of delivery, the representativeness of the sample and settings, and the feasibility of delivering the intervention as key components in translating research findings into practice. More recently, guides for intervention development, such as PRACTIS (PRACTical planning for Implementation and Scale-up) [59], outline an iterative multi-step process and considerations for the creation of interventions to more closely align with the prototypical characteristics of the population, setting, and context where an intervention is ultimately intended to be delivered [60].

Consideration for the elements represented in the scalability and implementation framework literature are paramount for the effective translation of interventions to improve population health. Discussions surrounding their importance, however, predominately focus on the middle to end of the translational pipeline continuum, largely ignoring the relevance of these issues during the early stages of developing and evaluating interventions in pilot studies. Frameworks that focus on pilot testing, such as ORBIT (Obesity-Related Behavioral Intervention Trials) [61], describe the preliminary testing of interventions to be done with “highly selected participants” under “ideal conditions” only to move on to more representative samples if the intervention reaches clinically or statistically significant targets under optimal conditions. This perspective aligns with the efficacy-to-effectiveness paradigm that dominates much of the behavioral intervention field, where interventions are initially studied under highly controlled conditions only to move to more “real-world” testing if shown to be efficacious [21]. These pilot testing recommendations are at odds with the scalability literature and the extensive body of work by Glasgow, Green and others that argues for a focus on evaluating interventions that more closely align with the realities of the conditions under which the intervention is ultimately designed to be delivered [49]. Hence, optimal conditions [24] may introduce external validity biases that could have a substantial impact on the early, pilot results and interpretation of whether an intervention should be tested in a larger trial [20–22, 55, 62].

The identification of generalizability biases may assist researchers to avoid the introduction of such artefacts in the early stages of evaluating an intervention and, in the long run, help to avoid costly and time-consuming decisions about prematurely scaling an intervention for definitive testing. Drawing from the scalability literature and incorporating key concepts of existing reporting guidelines, such as TIDieR [63], CONSORT [9], TREND [64], SPIRIT [65], and PRECIS-2 [51, 52] we describe the development of an initial set of risk of generalizability biases and provide empirical evidence regarding their influence on study level effects in a sample of published pilot studies that are paired for comparison with a published larger-scale efficacy/effectiveness trial of the same or similar intervention on a topic related to childhood obesity. The purpose of this study was to describe the rationale for generating an initial set of “risk of generalizability biases” (defined below) that may lead to exaggerated early discoveries [66] and therefore increase the risk of subsequent efficacy and effectiveness trials being unsuccessful. We provide empirical support of the impact of these biases using meta-analysis on outcomes from a number of published pilot studies that led to testing an intervention in a larger efficacy/effectiveness trial on a topic related to childhood obesity and provide recommendations for avoiding these biases during the early stages of testing an intervention.

## Methods

For this study, we defined behavioral interventions as interventions that target one or more actions individuals take that, when changed in the appropriate direction, lead to improvements in one or more indicators of health [67, 68]. Behavioral interventions target one or more behaviors in one of two ways – by directly targeting individuals or by targeting individuals, groups, settings or environments which may influence those individuals. Behavioral interventions are distinct from, but may be informed by, basic or mechanistic research studies that are designed to understand the underlying mechanisms that drive behavior change. Mechanistic studies are characterized by high internal validity, conducted in laboratory or clinical settings, and conducted without the intent or expectation to alter behavior outside of the experimental manipulation [69–72]. Thus, behavioral interventions are distinct from laboratory- or clinical-based training studies, pharmacological dose-response or toxicity studies, feeding and dietary supplementation studies, and the testing of new medical devices or surgical procedures.

We defined *“behavioral intervention pilot studies”* as studies designed to test the feasibility of a behavioral intervention and/or provide evidence of a preliminary effect(s) in the hypothesized direction [2, 10, 61]. These studies are conducted separately from and prior to a larger-scale, efficacy/effectiveness trial, with the results used to inform the subsequent testing of the same or refined intervention [61]. Behavioral intervention pilot studies, therefore, represent smaller, abbreviated versions or initial evaluations of behavioral interventions [10]. Such studies may also be referred to as “feasibility,” “preliminary,” “proof-of-concept,” “vanguard,” “novel,” or “evidentiary” [3, 6, 61].

### Study design

A systematic review was conducted for published studies that met our inclusion criteria (see below), with all reviews of database updated and finalized by December 31st, 2018. All procedures and outcomes are reported according to the PRISMA (Preferred Reporting Items for Systematic review and Meta-Analysis) [73] statement.

### Data sources and search strategy

A comprehensive literature search was conducted across the following databases: PubMed/Medline; Embase/Elsevier; EBSCOhost, and Web of Science. A combination of MeSH (Medical Subject heading), EMTREE, and free-text terms, and any boolean operators and variants of terms, as appropriate to the databases, were used to identify eligible publications. Each search included one or more of the following terms for the sample’s age - child, preschool, school, student, youth, and adolescent - and one of the following terms to be identified as a topic area related to childhood obesity - obesity, overweight, physical activity, diet, nutrition, sedentary, screen, diet, fitness, or sports.

To identify pairs of studies that consisted of a published pilot study with a larger, more well-powered trial of the same or similar intervention, the following procedures were used. To identify pilot studies, the following terms were used: pilot, feasibility, proof of concept, novel, exploratory, vanguard, or evidentiary. These terms were used in conjunction with the terms regarding sample age and topic area. To identify whether a pilot study had a subsequent larger, more well-powered trial published, the following was conducted. First, using a backwards approach, we reviewed published systematic reviews and meta-analyses on interventions targeting a childhood obesity-related topic that were published since 2012. The reviews were identified utilizing similar search terms as described above (excluding the pilot terms), with the inclusion of either “systematic review” or “meta-analysis” in the title/abstract. All referenced intervention studies in the reviews were retrieved and searched to identify if the study cited any preliminary pilot work that informed the intervention described and evaluated within the publication. Where no information about previous pilot work was made or statements were made about previous pilot work, yet no reference(s) were provided, contact via email with the corresponding author was made to identify the pilot publication.

All pilot studies included in the final sample for pairing with a larger, more well-powered trial required that the authors self-identified the study as a pilot by either utilizing one or more the terms commonly used to refer to pilot work somewhere within the publication (e.g., exploratory, feasibility, preliminary, vanguard), or the authors of a larger, more-well powered trial had to specifically reference the study as pilot work within the publication of the larger, more well-powered trial or protocol overview publication.

### Inclusion criteria

The following inclusion criteria were used: study included youth ≤18 years, a behavioral intervention (as defined previously) on a topic related to childhood obesity, have a published pilot and efficacy/effectiveness trial of the same or similar intervention, and were published in English. An additional inclusion criterion for the efficacy/effectiveness trials was the trial had to have a comparison group for the intervention evaluated. This criterion was not used for pilot studies, as some pilot studies could use a single group pre/post-test design.

### Exclusion criteria

Exclusion criteria were articles, either pilot or efficacy/effectiveness, that only provided numerical data associated with outcomes found to be statistically significant, reported only outcomes associated with compliance to an intervention, or the published pilot study only described the development of the intervention and did not present outcomes associated with preliminary testing/evaluation the intervention on one or more outcomes.

### Data management procedures

For each search within each database, all identified articles were electronically downloaded as an XML or RIS file and uploaded to Covidence (Covidence.org, Melbourne, Australia) for review. Within Covidence, duplicate references were identified as part of the uploading procedure. Once uploaded, two reviewers were assigned to review the unique references and identify those that met the eligibility criteria based on title/abstract. Where disagreements occurred, a third member of the research team was asked to review the disputed reference to make a final decision. Full-text PDFs were retrieved for references that passed the title/abstract screening. These articles were reviewed and passed on to the final sample of studies for the extraction of relevant study characteristics and outcomes. For included studies, all reported outcomes (e.g., means, standard deviations, standard errors, differences, change scores, 95% confidence intervals) were extracted for each study for analyses (described below).

### Defining and identification of risk of generalizability biases

Prior to reviewing the full-text articles that met the inclusion criteria, a candidate list of risk of generalizability biases was developed by the study authors, operationally defined, and their hypothesized influence on study outcomes determined based on the scalability, scaling-up, and dissemination/implementation of interventions for widespread uptake and population health impact [41–50] and pragmatic trial design [51–53] literature. After the initial set of risk of generalizability biases were developed and operationally defined, three reviewers (MB, KB, LD) independently reviewed the full-texts of the pilot and efficacy/effectiveness trial pairs for the potential presence of the biases. Each risk of generalizability bias was classified as either “present” or “absent”. Where discrepancies were identified, discussion regarding the evidence for bias was conducted to resolve the disagreement. In addition, during the review of the pilot and efficacy/effectiveness pairs, additional biases were identified, discussed, defined, and added to the list of risk of generalizability biases, where necessary. A total of 9 risk of generalizability biases were identified and operationally defined. Each bias, along with the definition, the hypothesized influence, and examples, are presented in Table 2.

**Table 2:** Operational Definitions of Risk of Generalizability Biases

Risk of Generalizability Bias	Questions to Ask	Increased Presence with Small Sample	Hypothesized Influence of the Presence of Risk of Generalizability Bias		Example	
			Pilot	Larger-Scale Efficacy/Effectiveness	Pilot	Larger-Scale Efficacy/Effectiveness
	What is the potential for difference(s) between…					
Intervention Intensity Bias	…the number and length of contacts in the current study and future evaluations of the intervention?	Yes	More frequent and longer contacts result in more effective intervention	Fewer and shorter contacts results in less effective intervention compared to pilot	19 lessons delivered (Salmon 2008 [34])^a^	6 lessons delivered (Salmon 2011 [37])^a^
Implementation Support Bias	…the amount of support provided to implement the intervention in the current study and future evaluations of the intervention?	Yes	Greater amounts of support to implement the intervention results in more effective intervention	Reduced support to implement the intervention results in less effective intervention compared to pilot	*“During the intervention, weekly, audio-taped debriefing meetings were held with the interventionists and project investigators to troubleshoot any problems with each session and to plan for the following sessions.”* (Beech 2003 [74])	
Intervention Delivery Agent Bias	…the level of expertise of the individual(s) who deliver the intervention in the current study compared to who will deliver the intervention in future evaluations?	Yes	Higher levels of expertise delivering the intervention results in more effective intervention	Lower level of expertise to deliver the intervention results in less effective intervention compared to pilot	*“…the programme was delivered by the researcher, a PE trained specialist, with extensive experience in the primary classroom.”* (Riley 2015 [75])	*“Classroom teachers were responsible for the planning and the delivery of all movement-based lessons during the intervention.”* (Riley 2016 [76])
Target Audience Bias	…the demographics of those that received the intervention in the current study to those who will receive the intervention in future evaluations?	No	Delivering intervention to more conducive, convenience sample or sample that is not representative of target population results in more effective intervention	Delivering intervention to sample of whom the intervention is intended results in less effective intervention compared to pilot	*“Although our sample size was... predominately white, and well-educated…”* (Sze 2015 [77])	
Intervention Duration Bias	…the length of the intervention provided in the current study to the length of the intervention in future evaluations?	No	Shorter duration results in more effective intervention	Longer duration less effective intervention compared to pilot	4-week intervention (Wilson 2005 [78])	17-week intervention (Wilson 2011 [79])
Setting Bias	…the setting where the intervention is delivered in the current study and the intervention delivery setting in future evaluations?	No	Delivering intervention in a more conducive, convenience location that is not representative of the target setting results in more effective intervention	Delivering intervention in a location more representative of target setting results in a less effective intervention compared to pilot	Intervention delivered on university campus ^b^	Intervention delivered in community setting ^b^
Measurement Bias	…the measures employed in the current study and the measures used in future evaluations of the intervention for primary/secondary outcomes?	Yes	Use of less reliable or valid measures of primary/secondary outcomes results in more effective intervention	Use of more reliable and valid measures results in less effective intervention compared to pilot	Pedometer used to measure physical activity (Lubans 2009 [80])	Accelerometer used to measure physical activity (Lubans 2012 [81])
Directional Conclusions	Are the intervention effect(s) in the hypothesized direction?	No	Less effective intervention	Reduces intervention effectiveness	*“The decline in physical activity among the participants was not anticipated…”* (Cliff 2007 [82])	
Outcome Bias	Is the primary outcome for future evaluations of the intervention measured in the current study?	No	Absences of measuring primary outcome results in more effective intervention	Absence of primary outcome collected in pilot results in less effective intervention tested in well-powered trial	Nutrients sold per day and number of items sold per day in school cafeterias (Hartstein 2008 [83])	Self-reported daily dietary intake of students (Siega-Riz 2011 [84])

^a^Although not labeled as a pilot study, the example illustrates the presence of the risk of generalizability bias in one study and altered in the subsequent trial

^b^Hypothetical example of the risk of generalizability bias as it could operate in a pilot to larger-scale efficacy/effectiveness trial

### Meta-analytical procedures

Standardized mean difference (SMD) effect sizes were calculated for each study across all reported outcomes. The steps outlined by Morris and DeShon [85] were used to create effect size estimates from studies using different designs across different interventions (independent groups pre-test/post-test; repeated measures single group pre-test/post-test) into a common metric. For each study, individual effect sizes and corresponding 95% CIs were calculated for all outcome measures reported in the studies.

To ensure comparisons between pilot and efficacy/effectiveness pairs were based upon similar outcomes, we classified the outcomes reported across pairs (i.e., pilot and efficacy/effectiveness trial) into seven construct categories that represented all the data reported [86]. These were measures of body composition (e.g. BMI, percent body fat, skinfolds), physical activity (e.g., moderate-to-vigorous physical activity, steps), sedentary behaviors (e.g., TV viewing, inactive videogame playing), psychosocial (e.g., self-efficacy, social support), diet (e.g., kcals, fruit/vegetable intake), fitness/motor skills (e.g., running, hopping), or other. For studies reporting more than one outcome within a category, for instance reporting five dietary outcomes in the pilot and reporting two dietary outcomes in the efficacy/effectiveness trial, these outcomes were aggregated at the construct level to represent a single effect size per construct per study using a summary calculated effect size and variance computed within Comprehensive Meta-Analysis (v.3.0). The construct-level was matched with the same construct represented within the pairs. For all comparisons, outcomes were used only if they were represented in both studies within the same construct as defined above. For instance, a study could have reported data related to body composition, diet, physical activity in both the pilot and efficacy/effectiveness trial, but also reported sedentary outcomes for the pilot only and psychosocial and fitness related outcomes for the efficacy/effectiveness only. In this scenario, only the body composition, diet, and physical activity variables would be compared across the two studies within the pair. Attempts were made at one-to-one identical matches of outcomes and reported units of the outcomes within pilot and efficacy/effectiveness pairs; however, there were numerous instances where similar constructs (e.g., physical activity, weight status) were measured in the pilot and efficacy/effectiveness study but were reported in different metrics across studies (e.g., steps in the pilot vs. minutes of activity in the efficacy/effectiveness or waist circumference in cm in the pilot and waist circumference in z-scores in the efficacy/effectiveness); therefore construct matching of the standardized effect size were used.

All effect sizes were corrected for differences in the direction of the scales so that positive effect sizes corresponded to improvements in the intervention group, independent of the original scale’s direction. This correction was performed for simplicity of interpretive purposes so that all effect sizes were presented in the same direction and summarized within and across studies. The primary testing of the impact of the biases was performed by comparing the changing in the SMD from the pilot study to the larger, efficacy/effectiveness trial for studies coded with and without a given bias present. All studies reported more than one outcome effect across the seven constructs (e.g., BMI outcomes and dietary outcomes); therefore, summary effect sizes were calculated using a random-effects multi-level robust variance estimation meta-regression model [87–89], with constructs nested within studies nested within pairs. This modeling procedure is distribution free and can handle the non-independence of the effects sizes from multiple outcomes reported within a single study.

### Criteria for evidence to support risk of generalizability biases

We examined the influence of the biases on the difference in SMD between the pilot and efficacy/effectiveness trials by testing the impact of each bias, separately, on the change in the SMD from the pilot to efficacy/effectiveness trial. All data were initially entered into Comprehensive Meta-Analysis (v.3.3.07) to calculate effect sizes for each reported outcome across constructs for all studies. The computed effect sizes, variances, and information regarding the presence/absence of the risk of generalizability biases were transferred into R (version 3.5.1) where a random-effects multi-level robust variance estimation meta-regression models were computed using the package “Metafor” [90].

Next, we examined whether the empirical evidence was in the hypothesized direction (see Table 2 for the biases and hypothesized directions). The final step was to examine the relationship between the presence of a bias and the sample size in the pilot and efficacy/effectiveness pairs. We hypothesized that the risk of generalizability biases would be more prevalent within smaller sized pilots. In pilot studies, a “small” sample size was classified as any pilot study with a total of 100 participants or less [91]. In absence of an established cutoff for efficacy/effectiveness trials, we defined a “small” sample size for the larger, more well-powered trials as any trial with 312 or fewer total participants. This size was based on the median sample size in the distribution of the sample in the identified well-powered trials.

## Results

**Fig. 1:**
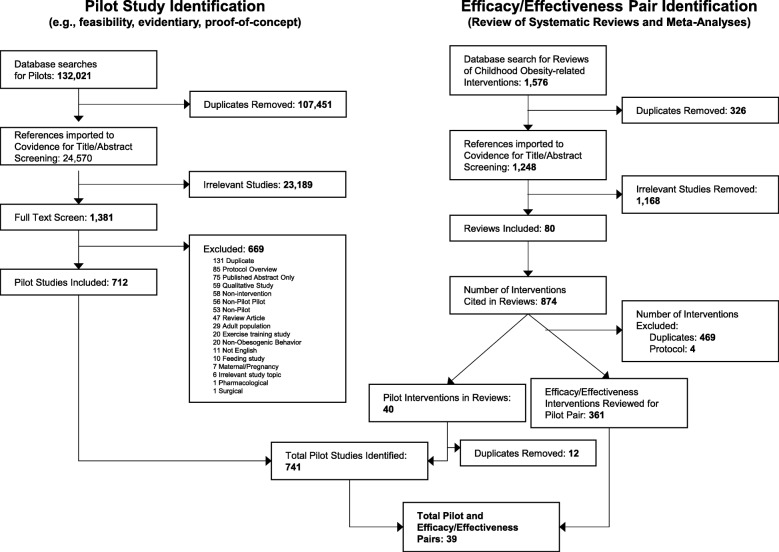
PRISMA diagram of literature search

A PRISMA diagram for the literature search is presented in Fig. 1. For the identification of published pilot studies, a total of 132,021 citations were identified across search engines and keywords, with 24,570 representing unique articles. After title/abstract and full-text screenings, a total of 741 articles met the final full text criteria as a pilot behavioral intervention on a topic related to childhood obesity. For the review of reviews, we identified a total of 1576 review studies. Of these, 80 reviews on a childhood obesity-related topic were identified that cited 362 unique efficacy/effectiveness interventions trials. After searching these interventions for reference to pilot work and cross-referencing the study authors with the identified pilot studies, we were able to confirm 42 pilots paired to 39 unique efficacy/effectiveness trials of the same or similar intervention [29, 74–84, 92–158]. Of these, one pilot and efficacy/effectiveness pair [94, 96] did not report similar outcomes across studies and therefore were not included in the analytical models. Three of the efficacy/effectiveness trials [84, 124, 136] had each published two separate pilot studies, reporting on different outcomes from the same pilot study [83, 100, 103, 123, 125, 159] on the same intervention evaluated in the efficacy/effectiveness publication and were included as pairs with a single efficacy/effectiveness trial and two pilots, each. Across all studies, a total of 840 individual effect sizes were initially computed, representing 379 effect sizes from the pilot studies and 461 from the efficacy/effectiveness trials. Aggregating at the construct level reduced the total individual effects to 182 across 38 pairs, with an average of 2.4 constructs represented within a pair (range 1 to 5).

**Fig. 2:**
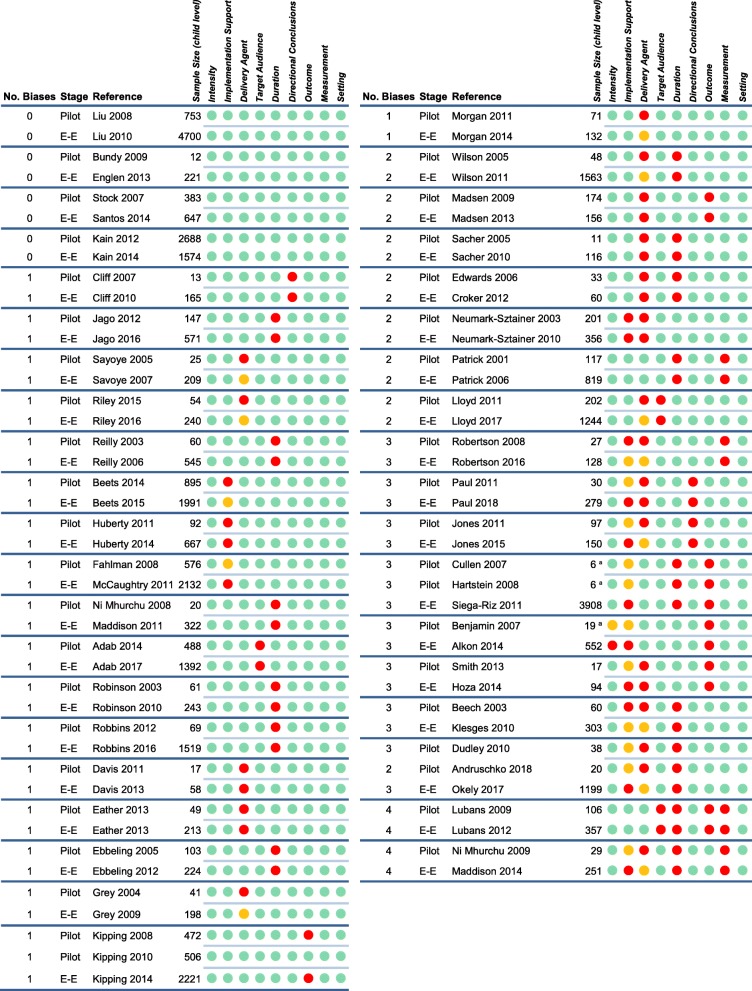
Presence of risk of generalizability biases in pilot and larger-scale efficacy/effectiveness pairs. Note: Red circle () indicates bias present, green circle () bias not present, orange circle () bias identified in pilot or well-powered but not the other. E-E = Efficacy/Effectiveness. ^a^ Sample size represents setting level (e.g., school, childcare) – child-level sample size not reported

The prevalence of the risk of generalizability biases across the 39 pilot and efficacy/effectiveness pairs are graphically displayed across each pair in Fig. 2. Overall, the most commonly observed biases were delivery agent bias (19/39 pairs), duration bias (15/39), implementation support bias (13/39), outcome bias (6/39), measurement bias (4/39), directional conclusion bias (3/39), and target audience bias (3/39). A single bias (setting bias) was not coded across any of the pairs, while intervention intensity bias was only identified once. In the review of 39 pairs, we found evidence of carry forward of two biases (i.e., bias present in both pilot and efficacy/effectiveness) – delivery agent bias and implementation support bias, with 8/39 of pairs coded as carrying forward delivery agent bias, while 4/39 carrying forward implementation support bias. Outcome bias was observed in 6/39, however, given the requirement of aligning constructs for analytical comparison, no analyses were conducted on this bias. This resulted in a total of six biases, of the nine, that had sufficient data for the analytical models.

**Fig. 3:**
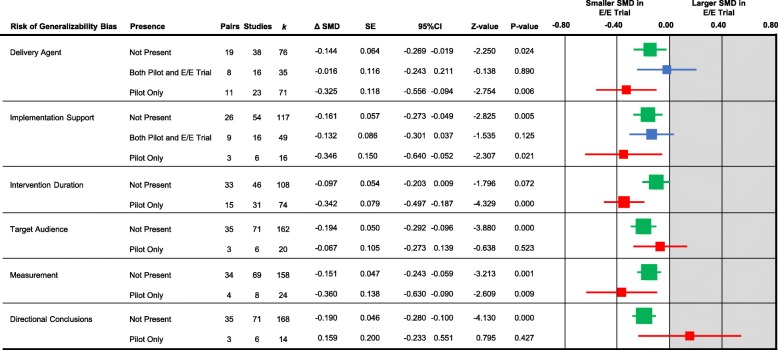
Forest plot of the change in the standardized mean difference (SMD) of the presence, absence, or carry forward of six risk of generalizability biases from a pilot to larger-scale efficacy/effectiveness (E/E) trial

The strength of evidence supporting the potential influence of each of the six biases are presented in Fig. 3. For four of the generalizability biases – delivery agent, implementation support, intervention duration, and measurement – the difference in the SMD (i.e., the larger, more well-powered trial SMD minus the pilot SMD) was larger in the pairs of pilot studies that had the bias present and subsequently did not have the bias present in the larger, more well-powered trials, compared to pairs that did not have the biases present. Specifically, the change in the SMD was − 0.325 (95CI − 0.556 to − 0.094) for agent delivery, − 0.346 (− 0.640 to − 0.052) for implementation support, − 0.342 (− 0.498 to − 0.187) for intervention duration, and − 0.360 (− 0.631 to 0.089) for measurement. Two biases, target audience (− 0.067, − 0.274 to 0.139) and directional conclusions (0.159, − 0.233 to 0.551), were not associated with major changes in the SMD. For pairs where biases that were coded as present in both the pilot and in the larger, more well-powered trials there was no major difference in the SMD for delivery agent (SMD = − 0.016, − 0.243 to 0.212), while a small reduction in the SMD was observed for implementation support (SMD = − 0.132 (− 0.301 to 0.037).

**Fig. 4:**
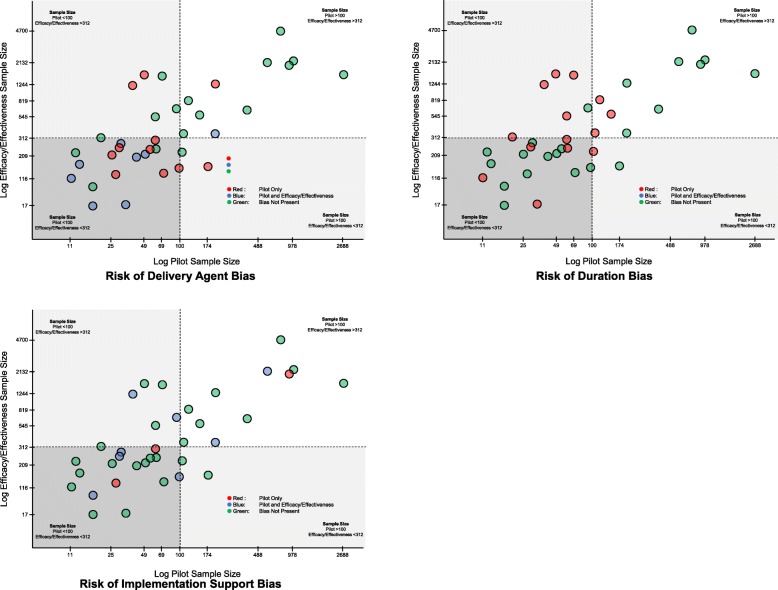
Association of the three most prevalent risk of generalizability biases with pilot and efficacy/effectiveness sample size. Note: The x- and y-axis represent the log of the total sample size per study. The tick marks represent the actual total sample size across the range of sample sizes in the studies.

The association of the presence of a bias with sample size of the pilot and efficacy/effectiveness pairs is presented in Fig. 4 for the three most prevalent biases (i.e., delivery agent, implementation support, and duration). Only 37 pairs were analyzed as two pairs [83, 84, 94, 96, 100] did not provide information on sample size at the child level, and therefore, could not be included in this analysis. Of the biases hypothesized to be influenced by smaller sample sizes, two demonstrated this pattern (i.e., implementation support and delivery agent, see Fig. 4). Of the 19 occurrences of delivery agent bias, 13 occurrences of implementation support bias, and 15 occurrences of intervention duration bias, these biases were coded in 16, 10, and 11 of the pairs with a pilot study classified as having a small sample size (*N* = 100 or less), respectively, [91].

## Discussion

The purpose of the current study was to define a preliminary set of risk of generalizability biases, specific to the early stages of testing of an intervention, provide a conceptual basis for their presence and to present evidence of their influence within a sample of pilot and the larger, more well-powered efficacy/effectiveness trial pairs on a topic related to childhood obesity. The identification of these biases should assist interventionists in avoiding the unintentional effects of biases related to external validity during the early stages of designing, conducting, and interpreting the outcomes from an intervention, as well as for reviewers of grants and manuscripts to determine whether the presence of one or more of the proposed biases may lead to exaggerated early discoveries [66] and subsequent failed efficacy/effectiveness trials.

In this study we identified 9 biases in pilot tested interventions that investigators, to a large extent, have control over whether or not they are introduced. These biases do not have to be introduced unless there is a strong and compelling rationale for their inclusion. One possible argument for including one or more of the risk of generalizability biases in a pilot (e.g., having a doctoral student deliver an intervention, testing the intervention over a short/abbreviated time period) are the resources available to conduct the study. Across the 39 pilot and efficacy/effectiveness pairs a total of 31 indicated the receipt of funding: 11 pilots were associated with NIH funding sources, 3 with sources from the National Institute for Health Research, 2 from the CDC, 11 from a foundation, and 4 from university or department/college level grants. “Well-funded” pilots, those with funding from the NIH, CDC or NIHR, contained biases at a similar rate as those considered to have lower amounts of funding (university/departmental award or foundation). Of the “well-funded” pilot studies, over 50% included risk of delivery agent bias, or risk of duration bias, while 42% included risk of implementation support bias.

While we could not confirm the total grant funding award for many of the pilot studies, of those where publicly available information was available, they received sizable awards to conduct the pilot study (e.g., NIH awards of R21 grants for 2 years and US$275,000 total direct costs). Interestingly, the resources to conduct a pilot, as evidenced by the receipt of federal grants, therefore, does not appear to be associated with the introduction or absence of a risk of generalizability bias. Thus, there must be alternative reasons that lead interventionists to include risk of generalizability biases in their pilot studies. At this time, however, it is unclear what rationale may be used for justifying the inclusion of risk of generalizability bias, particularly for those risk of generalizability biases that demonstrated the strongest relationship with differences in effect size estimations. Possible reasons may include the pressure to demonstrate initial feasibility and acceptability and potential efficacy which would then increase the chance of receiving funding for a larger study, the need for “statistically significant’ effects for publication, existing paradigms that endorse highly controlled studies prior to more real-world contexts or a combination of one or more of these reasons [24, 160, 161]. This may be a function of the pressures of securing grant funding for promotion or keeping a research laboratory operating [162].

With the creation of any new intervention there is a risk of it not being feasible, acceptable or potentially efficacious. Testing a new intervention on a small scale is a logical decision given the high-risk associated with the intervention not resulting in the anticipated effects [163]. Smaller scale studies are less resource intensive, compared to efficacy/effectiveness studies and thus, are a natural choice for pilot studies. It is also important to recognize that early “evidence of promise” from studies that may have design weaknesses is often used to secure further research funding and as such pilot studies often have in-built design limitations. Because a study is small in scale, it does not imply that the risks of generalizability biases described herein should be introduced. Our findings indicate, however, that a “small sample” size appears to serve as a proxy for the introduction of some of the biases that demonstrated the most influence on study level effects. This susceptibility to the biases, such as delivery agent bias and implementation support bias can, from a practical standpoint, operate more easily with smaller sample sizes. Interestingly, not all small sample pilot studies had evidence of delivery agent bias, implementation support bias, or duration bias, indicating small sample size studies can be conducted without the biases.

It is reasonable to assume that certain aspects of an intervention would (and at times should) be modified based upon the results of the pilot testing. Piloting an intervention affords this opportunity – the identification of potentially ineffective elements and their removal or the identification of missing components within an intervention that are theoretically and/or logically linked to the final interventions’ success in a larger-scale trial. If changes are necessary and, perhaps substantial, re-testing the intervention under pilot conditions (e.g., smaller sized study) is necessary. In fact, the ORBIT model calls for multiple pilot tests of an intervention to ensure it is ready for efficacy/effectiveness testing [61]. Within the sample of pilot and efficacy/effectiveness trial pairs, we identified many pilot studies whose findings suggested the next testing of the intervention should have been another pilot, instead of the larger-scale, efficacy/effectiveness trial identified. Part of the decision to move forward, despite evidence suggesting further refinement and testing of the refinements is necessary, could be attributed to incentives such as the need to secure future grant funding. In the efficacy/effectiveness literature, optimistically interpreting findings, despite evidence of the contrary, is referred to as “spin” [164, 165]. How such a concept applies to pilot studies is unclear and needs further exploration to whether “spin” is operating as a bias during the early stages of testing an intervention. Across our literature searches, we found no evidence of multiple pilot studies being conducted prior to the efficacy/effectiveness trial. Of the pilot to efficacy/effectiveness pairs that had two pilot studies published, these were pilot studies reporting different outcomes from the same pilot testing, rather than a sequential process of pilots. This suggests that published pilot studies, at least within the field of childhood obesity, are conducted only once, with interventionists utilizing the results (either positive or null) to justify the larger-scale evaluation of the intervention.

Our findings highlight that intervention researchers need to carefully consider whether information obtained from pilot tests of an intervention delivered by highly trained research team members, with extensive support for intervention delivery, over short timeframes with different measures than are to be used in the larger-trial can be sustained and is consistent with what is intended to-be-delivered in the efficacy/effectiveness trial. Including one or more of these biases in a pilot study could result in inflated estimates of effectiveness during the pilot and lead interventionists to believe the intervention is more effective than the actual effect achieved when delivered in a efficacy/effectiveness trial without these biases [14, 26, 166]. These are critical decisions because, if the purpose of a pilot study is to determine whether a large-scale trial is warranted, yet the outcomes observed from the pilot study are contingent upon the features included in the pilot that are not intended to be or cannot be carried forward in an efficacy/effectiveness trial, the likelihood of observing limited or null results in the efficacy/effectiveness trial is high. This scenario renders the entire purpose of conducting a pilot evaluation of an intervention a meaningless exercise that can waste substantial time and resources, both during the pilot and the larger-scale evaluation of an ineffective intervention.

Based on these findings, the following is recommended:

1. Carefully consider the impact of the risk of generalizability biases in the design, delivery, and interpretation of pilot, even in small sample size pilots and their potential impact on the decision to progress to a larger-scale trial
2. All pilots should be published, and efficacy/effectiveness studies should reference pilot work
3. When reporting pilot studies, information should be presented on the presence of the risk of generalizability biases and their impact on the outcomes reported discussed
4. When reviewers (e.g., grant, manuscript) review pilot intervention studies, evidence of the presence and impact of the risk of generalizability biases should be considered
5. If a pilot was “unsuccessful”, it should not be scaled-up but rather modified accordingly and re-piloted

Despite the initial evidence presented to support the utility of the risk of generalizability biases, there are several limitations that need to be considered. First, the sample in this study was limited to only 39 pilot and efficacy/effectiveness pairs, despite identifying over 700 published pilot and over 360 efficacy/effectiveness intervention studies. The publication of pilots, in addition to the clear reference to pilot work in efficacy/effectiveness studies needs to be made to ensure linkages between pilot and efficacy/effectiveness studies can be made. Second, a possibility exists that the over- or under-estimation of effects reported herein are also due to unmeasured biases, beyond the risk of generalizability biases investigated here, and thus, readers need to take this into consideration when evaluating the impact of the risk of generalizability biases. Third, the absence of a risk of generalizability bias does not infer that there was no bias. Rather, it simply refers to the inability to identify evidence in a published study of the presence of a given risk of generalizability bias. Hence, one or more of the risk of generalizability biases could have been present, yet not reported in a published study and therefore be undetectable. Fourth, it is possible that in the search we missed some pilot and larger-scale study pairs due to a lack of clear labeling of pilot studies. Finally, the evidence presented was only gathered from a single topic area – childhood obesity. It is unclear if the risk of generalizability biases exists and operate similarly within other intervention topics or if new risk of generalizability biases would be discovered that were not identified herein. Future studies need to explore this to develop an exhaustive list of recommendations/considerations for interventionists developing, testing, and interpreting outcomes from pilot intervention studies.

In conclusion, pilot studies represent an essential and necessary step in the development and eventual widespread distribution of public health behavioral interventions. The evidence presented herein indicates there are risk of generalizability biases that are introduced during the pilot stage. These biases may influence whether an intervention will be successful during a larger, more well-powered efficacy/effectiveness trial. These risk of generalizability biases should be considered during the early planning and design phase of a pilot and the interpretation of the results both for interventionists and reviewers of grants and scientific manuscripts. Thus, testing an intervention at the early stages under conditions that it would not be tested again may not provide sufficient evidence to evaluate whether a larger-scale trial is warranted. Future studies need to continue to refine and expand the list of risk of generalizability biases and evaluate their presence with study level effects across different social science and public health behavioral intervention topic areas.

## Data Availability

Access to the data will be made available upon completion of the entire project.
